# Wild edible plants collected by Hani from terraced rice paddy agroecosystem in Honghe Prefecture, Yunnan, China

**DOI:** 10.1186/s13002-019-0336-x

**Published:** 2019-11-27

**Authors:** Binsheng Luo, Bo Liu, Hongzhen Zhang, Hongkang Zhang, Xuan Li, Lijuan Ma, Yizhou Wang, Yujia Bai, Xinbo Zhang, Jianqin Li, Jun Yang, Chunlin Long

**Affiliations:** 1 College of Life and Environmental Sciences, Minzu University of China, Beijing, 100081 China; 2 Key Laboratory of Ethnomedicine (Minzu University of China), Ministry of Education, Beijing, 100081 China; 3 Bureau of Word Heritage of Honghe Prefecture, Mengzi, 661100 Yunnan China; 4 Kunming Institute of Botany, Chinese Academy of Sciences, Kunming, 650201 China

**Keywords:** Hani terraced rice paddy fields, Wild edible plants, Ethnobotany, Hani ethnic group

## Abstract

**Background:**

The Hani people in the Honghe Prefecture of Southeastern Yunnan, China, have practiced terraced rice paddy farming for more than 1300 years. These rice fields, combined with the surrounding forests and water systems, form a special agroecosystem that has attracted both tourists and scientists. For centuries, the local people have traditionally collected wild edible plants (WEP) from the agroecosystem, but this unique traditional practice in this area has never been reported.

**Methods:**

Ethnobotanical fieldwork was conducted in four counties (Yuanyang, Honghe, Jinping, and Lüchun) between 2014 and 2019. Local self-identified Hani people (186) were interviewed, and information concerning local WEP species was obtained, documented, and analyzed. Plant samples and voucher specimens were collected for taxonomic identification.

**Results:**

A total of 224 WEP species, belonging to 90 families and 170 genera, were recorded as used by the Hani people in Honghe. The most common WEP parts used include fruits, stems, and leaves, and the most common preparation methods include eating as a potherb (wild vegetable) and eating fresh. Some WEPs, like *Phyllanthus emblica* and *Dioscorea subcalva*, have unique preparation methods. The use-value (UV) and frequency of utilization index (FUI) of WEP species were analyzed. The 20 WEP species with the highest UV were noted as particularly important to the Hani people’s daily life in Honghe.

**Conclusion:**

A large majority of these WEP species possess tremendous economic potential for future development. However, the diversity of WEP species, the associated traditional knowledge, and the broader agroecosystem are facing challenges such as biodiversity loss and pollution from chemical pesticides and fertilizers. This study may help local people to recognize the value of local WEP species and associated traditional knowledge, as well as provide ethnobotanical information for the future development of this tourism region.

## Background

The terraced rice paddy fields of the Hani people of Southeastern Yunnan, China, represent a unique agroecosystem with significant economic, ecological, and esthetic values [1]. Due to the dramatic altitudinal range in this area (144–2939 m) [2], there is a significant diversity of climatic zones and associated micro-climates [3]. These climatic zones, in order from low to high altitude, are southern subtropical, middle subtropical, northern subtropical, warm temperate, temperate, and cold temperate climates [3]. This complex topography and diversity of climates significantly contribute to the richness of local biodiversity [4].

Since the 1960s, the Hani terraced rice paddy fields have attracted scientific interest, and they have even been elected into the Globally Important Agricultural Heritage Systems (2010) and the UNESCO World Heritage List (2013) [5]. For example, Zhu et al. [6] carried out a series of experiments in the terraced rice paddy fields in Honghe Hani and found that crop heterogeneity could solve the vulnerability of monoculture crops to disease; Li et al. [7] studied the agricultural soils by molecular methods and revealed the dynamics of organic matter in Yuanyang Terrace. However, there have been no studies on the wild edible plants (WEP species) collected and consumed by the Hani people in Honghe Hani terraced rice paddy system. In addition to scientific interest, the Honghe rice terraces have attracted more than 20 million tourists since 2014 [8]. Due to their interests in local foods, tourists have driven a demand for WEP species on the menus of local restaurants.

The Hani people speak their own language, which does not have a traditional writing system. After 1957, a set of writing characters of the Hani language based on Latin was invented with the help of the Chinese government and linguists [8].

**Fig. 1:**
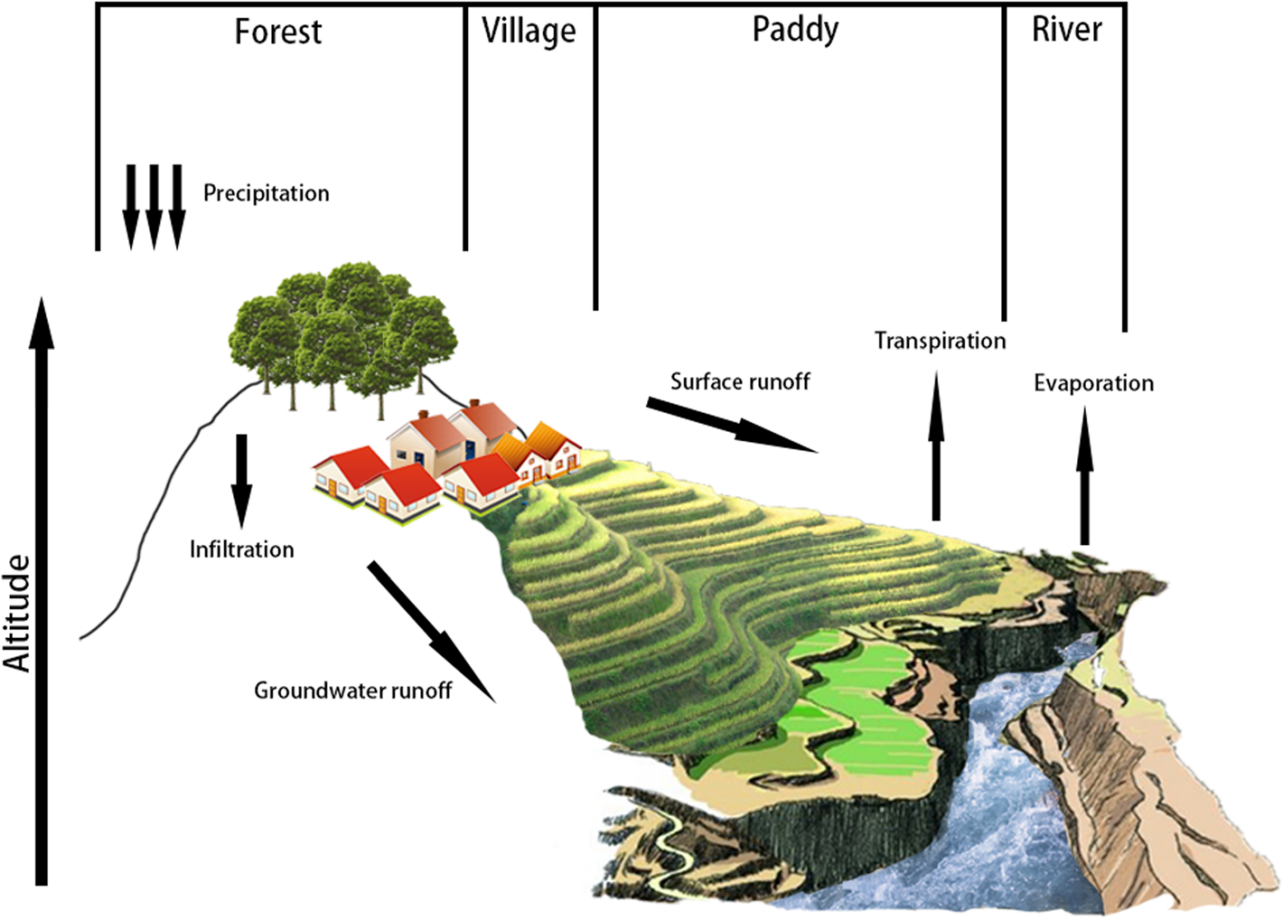
The construction of the Hani terraced rice paddy field agroecosystem

About 1300 years ago [9], the Hani people migrated to Southeast Yunnan and began the cultivation of rice paddies in terraced hillside fields, forming a sustainable agroecosystem consisting of four major components: forests, villages, terraced rice paddies, and river systems (Fig. 1) [10, 11]. The evergreen forests control the water in the soil, acting like a natural reservoir to maintain the water year long, and also provide water for the villages and terraced rice paddies in lowlands through water channels built by Hani people [10]. Also, the hot and humid valley climate frequently generates a thick fog that helps to maintain moist air throughout the year. This unique four element–based Hani agroecosystem ensures a stable water supply. Consequently, the Hani terraced rice paddy fields did not suffer any significant damage from the historic 2009–2010 drought in China [2, 12].

Due to this unique and complex agroecosystem, together with its rich biodiversity, much traditional ethnobotanical knowledge has been developed and accumulated by the local people, especially knowledge about WEP species. Since WEP species have not been domesticated and grown on a large scale, they must be obtained from the natural environment in order to be used as food [13, 14].

In some parts of Yunnan, there has been a rapid replacement of complex agroecosystems by intensive monocultures of commercial crops, such as bananas, which is often accompanied by the use of inorganic herbicides and pesticides [15]. Rising awareness and concern about the possible health effects of pesticides and herbicides on human health has dramatically increased demand for organic foods in China [15]. This interest extends to WEP species as they are wild harvested. For example, many WEP species have made their way into ethnic minority recipes found on high-end restaurant menus. Consequently, some WEP species have tremendous market potential and are particularly popular in tourist areas because of their perceived advantages of being pesticide-free, naturally grown, high in nutrients, and fresh in taste [13, 16]. According to a Web of Science search of bibliometric and mapping knowledge domains, WEP species have always been an essential hotspot in ethnobotanical research [17]. WEP species in the Hani terraced rice paddy agroecosystem are often used to supplement daily food resources or to help to overcome seasonal food shortages [18]. Additionally, some WEP species possess medicinal properties that may help protect indigenous people against diseases [18, 19].

Although the Hani people have lived in the Honghe Hani terraced rice paddy system for centuries, their traditional knowledge and associated biodiversity are rapidly being lost due to socio-economic changes and access to modern technologies [4, 11]. Consequently, decreasing traditional knowledge will likely lead to a decrease in biodiversity, especially the diversity of WEP species [20]. Therefore, saving local traditional knowledge and protecting biodiversity are urgent [21]. To our knowledge, no previous studies have documented the WEP species in Hani terraced rice paddy agroecosystems. Thus, this investigation on the WEP species in Hani was conducted. This study recorded traditional knowledge of WEP species, which may protect it from disappearing in a rapid-developing era. The related research results may also provide scientific guidance for WEP species consumption, information of economic benefit to local communities for future sustainable development, and application of WEP species.

## Methods

### Study area

**Fig. 2:**
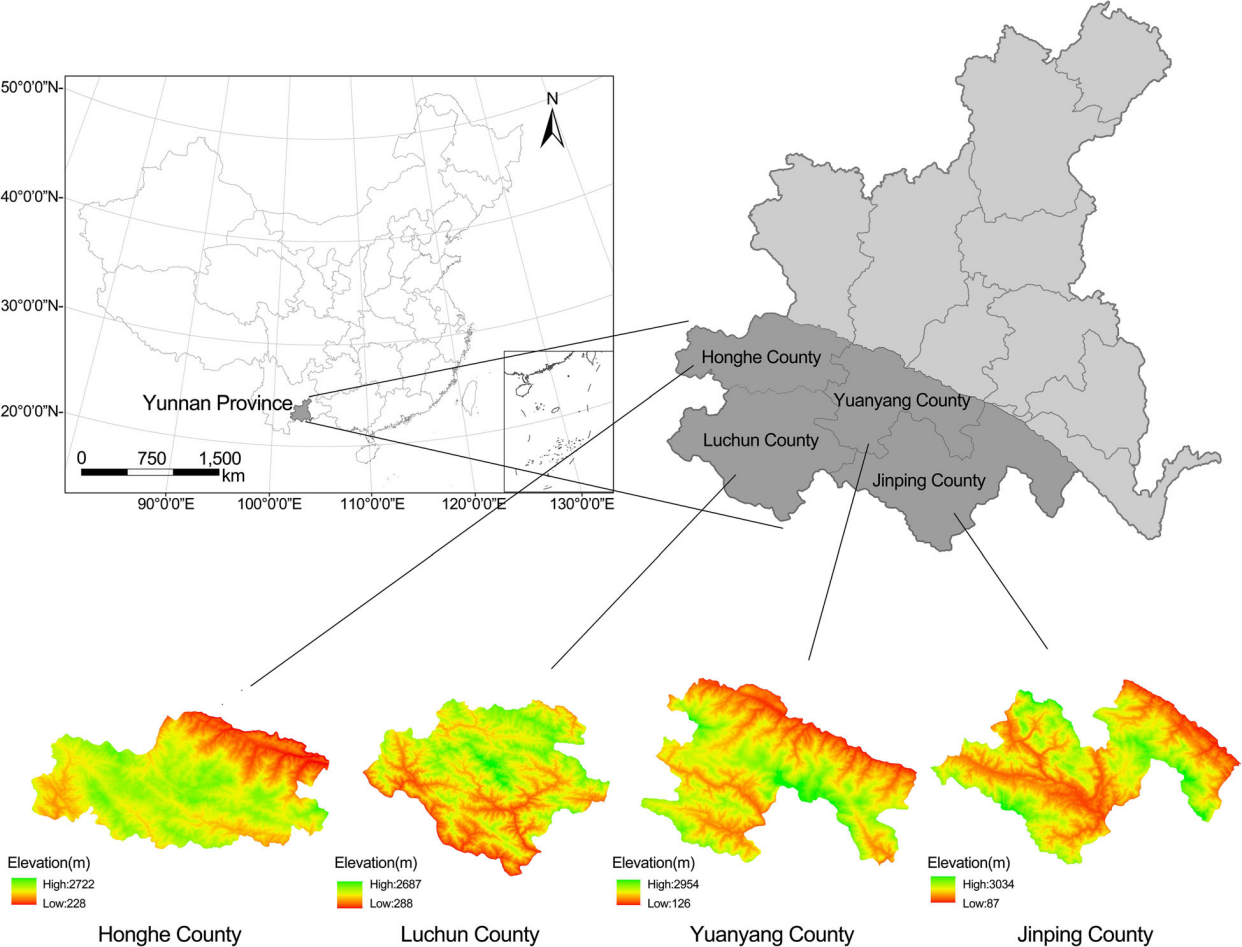
Location and terrain of the study area

Before the field survey, a literature review was conducted to obtain information about the region of Hani terraced rice paddy fields, including climates, topography, vegetation types, and culture [22]. During 2014–2019, ethnobotanical studies were carried out in four counties (Yuanyang, Lüchun, Honghe, and Jinping), which cover more than 47,000 ha, including most of the area containing Hani terraced rice paddy fields (Fig. 2) [23]. All study sites and their visit times are recorded in detail in Table 1. In consideration of local landscape diversity, this investigation was conducted in almost every landscape of this agroecosystem, including farming areas, forests, villages, home gardens, and water source areas [23]. Additionally, local markets in different villages and counties were surveyed repeatedly, as the markets often reflect the wide variety of local knowledge in daily life [24].

**Table 1:** Study sites in Honghe Hani terraced rice paddy system

County name	Village and township	Times visited
Honghe County	Lüshuge Village, Jiayin Township	2
Honghe County	Lonajia Village, Jiayin Township	1
Honghe County	Baohua Township	2
Jinping County	Xiongjia Village, Adebo Township	2
Jinping County	Shuiyan Village, Ma’andi Township	2
Lüchun County	Lüchun County	2
Lüchun County	Lagu Village, Sanmeng Township	1
Yuanyang County	Xiaoxinjie Township	1
Yuanyang County	Niujiaozhai Township	1
Yuanyang County	Qingkou Village, Xinjie Township	1
–	The junction of Lüchun County and Yuanyang County	1

### Data collection, voucher specimen collection, and data analysis

**Fig. 3:**
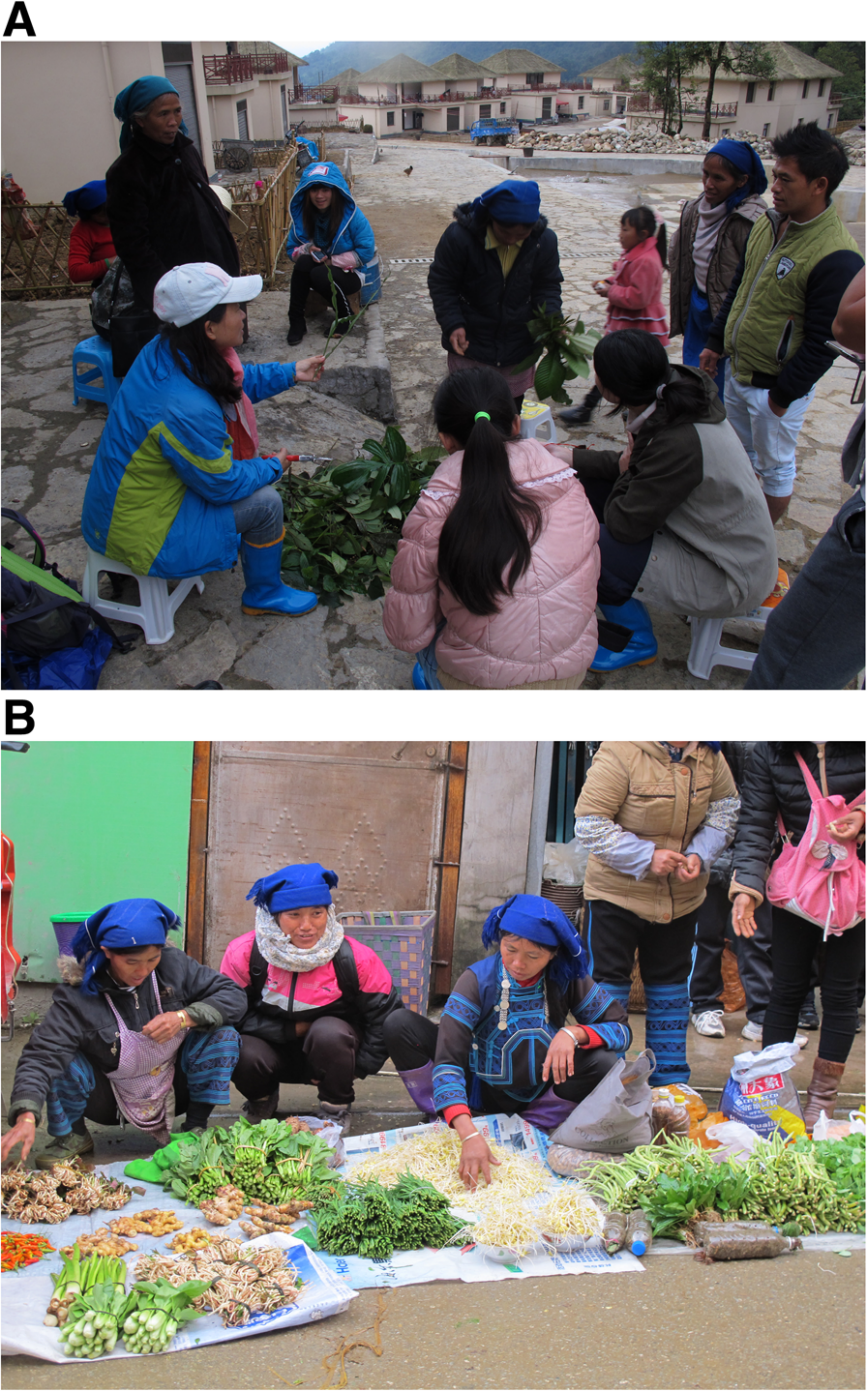
Focal group discussion (**a**) and Hani women in a local market (**b**)

A variety of different ethnobotanical and social science methods were used to collect data about the WEP 7species in this region. These methods included participatory rural appraisal (PRA), direct observation, semi-structured interviews, key informant interviews, and focal group discussions (Fig. 3) [4, 25, 26]. In total, 186 native Hani people, including 160 people older than 50 years of age, were interviewed. Seventy of them were male, and 116 were female. They were mostly local farmers, and many of them collected WEPs to sell in local markets. The primary content of the interview consisted of “5 W + H” questions (i.e., questions concerning what, when, where, who/whom, why, and how the subjects utilize WEP) [24, 26, 27]. With the assistance of Hani local experts, voucher specimens were gathered from different habitats around the study sites. Plant species were identified by Dr. Chunlin Long, Dr. Bo Liu, and Ms. Jun Yang. The voucher specimens were deposited at the College of Life and Environmental Sciences at the Minzu University of China in Beijing.

The data collected in the Honghe area was collated into an inventory containing all the WEP species and related information. The use-value (UV) of each WEP was calculated to evaluate the relative importance of each plant based on the number of times cited and the number of informants [28, 29]. The formula for UV is

$$ \mathrm{UV}\kern0.33em =\kern0.33em \left(\sum {U}_i\right)\kern0.33em /\kern0.33em N $$

*U*_i_ is the times cited by each informant for a certain WEP, while *N* is the total number of informants [29]. The frequency of utilization index (FUI) of WEP species was graded according to the frequency of consumption by local people. FUI can also reflect the degree of closeness between WEP species and the local community [29]. The FUI scores range from 0 to 5 and varied by the consumption frequency (Table 2) [29].

**Table 2:** The FUI value and corresponding category

Consumption frequency	FUI
More than once a week	5
Once a week	4
Once a month	3
More than once a year, less than once a month	2
Once a year	1
No consumption in last 30 years	0.5

## Results and discussion

### Diversity of WEP species in Honghe terraced rice paddy fields

Based on our investigations, 224 WEP species belonging to 170 genera and 90 families, along with related information such as scientific names, family names, life forms, vernacular names, edible parts, and processing methods, were documented (Table 3). According to the recorded WEP species, more than half of the species are woody plants (50.9%), including trees (30.4%) and shrubs (20.5%). There were 80 species of herbaceous plants (35.3%), 21 species of lianas (9.4%), and 10 species of bamboos (4.5%) (Table 4).

**Table 3:** Inventory of WEP species in Honghe terraced rice paddy system

Scientific name	Vernacular Name	Life form	Family name	Parts used	Preparation and uses	Study sites	Voucher number	FUI	UV
Gymnospermae									
*Gnetum montanum* Markgr.	Wo ni ai xi	Liana	Gnetaceae	Seed	Cooked thoroughly and eaten (kernel)	Lüchun County	201,606–19	0.6	0.21
*Gnetum pendulum* C.Y.Cheng	Mang dao	Liana	Gnetaceae	Seed	Cooked thoroughly and eaten (kernel)	Lagu Village, Sanmeng Township	HHD-31	0.5	0.16
Angiospermae									
*Kadsura coccinea* (Lem.) A.C.Sm.	Hei lao hu	Liana	Schisandraceae	Fruit	Ripe fruits are eaten fresh	Lüshuge Village, Jiayin Township	201,610–04	2.4	0.44
*Houttuynia cordata* Thunb.	Pa huo	Herb	Saururaceae	Rhizome	Potherb or flavoring agent	Lagu Village, Sanmeng Township	HHD-54	4.5	0.89
*Piper betle* L.	Fa qie wei niu	Liana	Piperaceae	Leaf	Flavoring agent	Lonajia Village, Jiayin Township	201,506–39	2.7	0.55
*Michelia hedyosperma* Y.W.Law	Ma la	Tree	Magnoliaceae	Seed	Flavoring agent	Lagu Village, Sanmeng Township	HHD-39	2.8	0.44
*Alphonsea mollis* Dunn		Tree	Annonaceae	Fruit	Ripe fruits are eaten fresh	Lonajia Village, Jiayin Township	201,506–35	2.0	0.57
*Litsea akoensis* var. *sasakii* (Kamik.) J.C. Liao		Tree	Lauraceae	Fruit	Flavoring agent	Lüchun County	201,606–26	2.4	0.54
*Litsea cubeba* (Lour.) Pers.	Mo ye la pi	Shrub	Lauraceae	Fruit	Flavoring agent	Shuiyan Village, Ma’andi Township	201,511–12	2.0	0.54
*Litsea pungens* Hemsl.	Si bi a si	Tree	Lauraceae	Fruit	Flavoring agent	Baohua Township	201,511–43	4.9	0.92
*Acorus gramineus* Aiton	Ji xiang	Herb	Acoraceae	Leaf, rhizome	Flavoring agent	Shuiyan Village, Ma’andi Township	201,511–13	2.8	0.46
*Amorphophallus konjac* K.Koch	Jia mo	Herb	Araceae	Tender leaf, tuber	Making “tofu”	Niujiaozhai Township	201,606–09	2.9	0.43
*Colocasia gigantea* (Blume) Hook. f.	Bo ju	Herb	Araceae	Petiole	Potherb (cooked thoroughly)	Xiaoxinjie Township	201,506–06	4.0	0.80
*Sagittaria trifolia* L.	Wo qi	Herb	Alismataceae	Tender leaf, rhizome	Potherb (stewed or stir-fried)	Lüshuge Village, Jiayin Township	201,610–24	2.4	0.46
*Dioscorea cirrhosa* Lour.	Ai la ma a si	Liana	Dioscoreaceae	Tuber	Cereal substitute in famine time	Xiaoxinjie Township	201,506–05	2.4	0.43
*Dioscorea subcalva* Prain et Burkill	Mo mo mang	Liana	Dioscoreaceae	Tuber	Making “tofu” (similar to konjac tofu)	Lagu Village, Sanmeng Township	HHD-45	2.6	0.46
*Heterosmilax yunnanensis* Gagnep.	Guo ge niao, a guo guo ne	Shrub	Smilacaceae	Tender leaf	Potherb (blanched in boiled water, then soaked in cold water for days. Usually stir-fried or made into soup)	The junction of Lüchun County and Yuanyang County	201,506–08	2.7	0.47
*Caryota urens* L.	Ha da a bo	Tree	Arecaceae	Flower	Snack (inflorescence sap is sweet)	Lüshuge Village, Jiayin Township	201,610–13	0.9	0.20
*Commelina benghalensis* Forssk.	A wei ya mo	Herb	Commelinaceae	Tender Leaf, tender stem	Potherb (boiled for 5–10 min, then soaked in water to debitterize)	Lüchun County	LB-27	2.0	0.49
*Commelina diffusa* Burm.f.	Nuo niu pao	Herb	Commelinaceae	Whole plant	Potherb (usually stewed with pork)	Lüshuge Village, Jiayin Township	201,610–15	2.9	0.42
*Streptolirion volubile* Edgew.	Mo dui dui han	Herb	Commelinaceae	Tender stem, leaf	Potherb (made into soup)	Xiaoxinjie Township	LB-16	2.2	0.56
*Monochoria vaginalis* (Burm.f.) C.Presl	Mi zuo wa, a bei bei za, e za, e bi ra	Herb	Pontederiaceae	Stem and leaf	Potherb	Lagu Village, Sanmeng Township	HHD-48	2.0	0.47
*Musa acuminata* Colla	Ruo a pao ruo a wo	Herb	Musaceae	Fruit, flower, pith part	Fruit: eaten fresh; flower and pith part: cooked as potherb	Baohua Township	201,511–44	3.8	0.70
*Musa itinerans* Cheesman		Herb	Musaceae	Flower, young bract	Potherb	Xiaoxinjie Township	LB-14	2.6	0.41
*Amomum maximum* Roxb.	Sa jia hong bi	Herb	Zingiberaceae	Fruit	Flavoring agent	Lüshuge Village, Jiayin Township	201,610–12	2.7	0.52
*Hedychium coronarium* J.Koenig	A ci a ye	Herb	Zingiberaceae	Flower, shoot	Potherb (usually stewed or stir fried)	Lonajia Village, Jiayin Township	201,506–53	2.3	0.45
*Zingiber striolatum* Diels		Herb	Zingiberaceae	Flower	Potherb	Lüshuge Village, Jiayin Township	201,610–22	3.0	0.73
*Acidosasa hirtiflora* Z.P.Wang and G.H.Ye		Bamboo	Poaceae	Shoot	Bamboo shoots	Shuiyan Village, Ma’andi Township	201,511–20	0.1	0.12
*Chimonobambusa yunnanensis* Hsueh et W.P. Zhang		Bamboo	Poaceae	Shoot	Bamboo shoots	Shuiyan Village, Ma’andi Township	201,511–22	1.0	0.18
*Chimonocalamus longiligulatus* Hsueh and T.P.Yi	Ha bo	Bamboo	Poaceae	Shoot	Bamboo shoots	Xiaoxinjie Township	LB-07	0.5	0.18
*Dendrocalamus membranaceus* Munro	A ha a bi	Bamboo	Poaceae	Shoot	Bamboo shoots	Xiongjia Village, Adebo Township	HHD-015	0.6	0.17
*Dendrocalamus peculiaris* Hsueh and D.Z.Li		Bamboo	Poaceae	Shoot	Bamboo shoots	Shuiyan Village, Ma’andi Township	201,511–23	0.8	0.25
*Indosasa singulispicula* T.H.Wen		Bamboo	Poaceae	Shoot	Bamboo shoots	Lonajia Village, Jiayin Township	201,506–43	0.1	0.15
*Indosasa sinica* C.D.Chu and C.S.Chao	A xiu a bo	Bamboo	Poaceae	Shoot	Bamboo shoots	Shuiyan Village, Ma’andi Township	201,511–17	0.6	0.06
*Melocalamus arrectus* T.P.Yi	A ha a bo	Bamboo	Poaceae	Shoot	Bamboo shoots	Shuiyan Village, Ma’andi Township	201,511–18	0.6	0.09
*Phyllostachys nigra* var. *henonis* (Mitford) Rendle	A mao a bo	Bamboo	Poaceae	Shoot	Bamboo shoots	Shuiyan Village, Ma’andi Township	201,511–21	0.9	0.04
*Schizostachyum funghomii* McClure	A che	Bamboo	Poaceae	Shoot	Bamboo shoots	Shuiyan Village, Ma’andi Township	201,511–19	0.5	0.25
*Akebia trifoliata* (Thunb.) Koidz.		Liana	Lardizabalaceae	Fruit	Ripe fruits are eaten fresh	Lüshuge Village, Jiayin Township	201,610–05	1.9	0.54
*Parabaena sagittata* Miers	Hua na wei niu	Liana	Menispermaceae	Leaf	Potherb	Shuiyan Village, Ma’andi Township	201,511–28	2.5	0.55
*Mahonia bealei* (Fortune) Pynaert	Shi shi, sou shou	Shrub	Berberidaceae	Fruit, stem	Stem: liquor brewing; fruit: eaten fresh	Lonajia Village, Jiayin Township	201,506–45	2.6	0.52
*Helicia nilagirica* Bedd.	Kong bai a bo	Tree	Proteaceae	Seed	Cooked seeds are used as grain substitute	Shuiyan Village, Ma’andi Township	201,511–01	2.7	0.58
*Dillenia indica* L.	Xi shi a di	Tree	Dilleniaceae	Fruit	Ripe fruits are eaten fresh	Qingkou Village, Xinjie Township	201,506–13	2.8	0.41
*Acacia pennata* (L.) Willd.	Tuo bo ji niu	Liana	Fabaceae	Tender leaf	Potherb	Baohua Township	HHD-25	3.4	0.65
*Bauhinia acuminata* L. var. *candida* (Roxb.) Voigt	Du bie a lo	Shrub	Fabaceae	Flower, young pod, seed, tender leaf	Potherb, seeds: cooked throughly and eaten (kernel)	Xiaoxinjie Township	LB-17	2.9	0.40
*Chamaecrista mimosoides* (L.) Greene		Herb	Fabaceae	Tender leaf	Tea substitute	Qingkou Village, Xinjie Township	201,506–19	0.5	0.14
*Chamaecrista nictitans* (L.) Moench subsp. *patellaris* (DC. ex Collad.) H. S. Irwin et Barneby var. *glabrata* (Vogel) H. S. Irwin et Barneby		Herb	Fabaceae	Tender leaf, tender stem	Tea substitute	Lüchun County	LB-18	1.2	0.30
*Gleditsia sinensis* Lam.	A si ni ma a hong	Tree	Fabaceae	Tender leaf	Potherb	Lüshuge Village, Jiayin Township	201,610–20	2.7	0.48
*Parochetus communis* D.Don	A wo la qian	Herb	Fabaceae	Flower	Potherb (stir-fried)	Lüchun County	201,606–31	2.5	0.57
*Senna tora* (L.) Roxb.		Herb	Fabaceae	Flower, leaf, young fruit, seed	Potherb, seed: substitute of coffee	Lüchun County	LB-28	2.6	0.48
*Tadehagi triquetrum* (L.) H.Ohashi	Qian ka a bo	Shrub	Fabaceae	Tender leaf, tender stem	Tea substitute	Lagu Village, Sanmeng Township	HHD-43	0.7	0.23
*Tamarindus indica* L.	Bi qian a si	Tree	Fabaceae	Fruit, tender leaf	Fruit: eaten fresh or made into compote; tender leaf: potherb (blanched before cooking)	Niujiaozhai Township	201,606–08	2.5	0.56
*Fagopyrum dibotrys* (D.Don) H.Hara	A za ca sa	Herb	Polygonaceae	Root	Potherb (usually made into soup)	Xiongjia Village, Adebo Township	201,506–04	2.2	0.57
*Polygala fallax* Hemsl.	Ha pa ha ma	Shrub	Polygalaceae	Flower, tender leaf	Potherb (usually made into soup)	Lonajia Village, Jiayin Township	201,506–52	2.3	0.47
*Polygonum cupitatum* Buch.-Ham. ex D.Don	A za za ni	Herb	Polygonaceae	Tender leaf	Potherb (usually made into soup)	Xiongjia Village, Adebo Township	201,506–03	2.0	0.46
*Polygonum hydropiper* L.	An ji ba qian	Herb	Polygonaceae	Tender leaf, tender stem	Potherb	Qingkou Village, Xinjie Township	201,506–24	2.8	0.54
*Polygonum molle* D. Don	Qian ge a si	Shrub	Polygonaceae	Fruit	Ripe fruits are eaten fresh	Baohua Township	201,511–33	2.2	0.50
*Polygonum perfoliatum* L.	A qian la qian a pa	Herb	Polygonaceae	Fruit	Ripe fruits are eaten fresh	Xiaoxinjie Township	LB-03	2.1	0.58
*Reynoutria japonica* Houtt.	Suan gan tong	Herb	Polygonaceae	Tender stem	Potherb	Lonajia Village, Jiayin Township	201,506–37	2.0	0.58
*Xanthophyllum yunnanense* C.Y. Wu		Tree	Polygalaceae	Fruit	Ripe fruits are eaten fresh	Lüshuge Village, Jiayin Township	201,610–07	2.7	0.42
*Crataegus pinnatifida* Bunge	Si pu a si	Tree	Rosaceae	Fruit	Ripe fruits are eaten fresh	Baohua Township	201,511–37	1.8	0.38
*Fragaria vesca* L.	O luo jia ba a si	Herb	Rosaceae	Fruit	Ripe fruits are eaten fresh	Lüshuge Village, Jiayin Township	201,610–08	2.1	0.44
*Pyrus calleryana* Decne.	Si peng a si	Tree	Rosaceae	Flower, fruit	Fruit: eaten fresh; flower: potherb (soaked in water to dibitterize, then stir-fried, made into soup or salad)	Lonajia Village, Jiayin Township	201,506–41	2.0	0.54
*Pyrus xerophila* T.T.Yu	A pei pei zi zuo	Tree	Rosaceae	Fruit	Ripe fruits are eaten fresh	Lüchun County	201,606–22	2.7	0.49
*Rubus ellipticus* var. *obcordatus* (Franch.) Focke	Huo wo	Shrub	Rosaceae	Fruit	Ripe fruits are eaten fresh	Lüchun County	201,606–21	2.7	0.38
*Rubus multibracteatus* H. Lév. and Vaniot		Shrub	Rosaceae	Fruit	Ripe fruits are eaten fresh	Lagu Village, Sanmeng Township	HHD-33	1.9	0.58
*Rubus parvifolius* L.	A guo luo a bei	Shrub	Rosaceae	Fruit	Ripe fruits are eaten fresh	Qingkou Village, Xinjie Township	201,506–15	2.5	0.56
*Elaeagnus conferta* Roxb.	Ba pen luo niu	Shrub	Elaeagnaceae	Fruit	Ripe fruits are eaten fresh	Lonajia Village, Jiayin Township	201,506–30	2.5	0.41
*Artocarpus lacucha* Buch.-Ham. ex D. Don	A niao niao bei	Tree	Moraceae	Fruit	Ripe fruits are eaten fresh	Lüshuge Village, Jiayin Township	201,610–09	2.9	0.57
*Artocarpus tonkinensis* A.Chev. ex Gagnep.	Ci gan gan nü	Tree	Moraceae	Fruit	Ripe fruits are eaten fresh	Lonajia Village, Jiayin Township	201,506–29	2.3	0.47
*Broussonetia papyrifera* (L.) L’Her.ex Vent.	Ma san	Tree	Moraceae	Flower, tender leaf	Potherb	Niujiaozhai Township	201,606–16	2.0	0.44
*Ficus auriculata* Lour.	Mu gua cai	Tree	Moraceae	Fruit	Ripe fruits are eaten fresh	Lüchun County	201,606–23	3.4	0.68
*Ficus hederacea* Roxb.	Jia ni ni bai	Shrub	Moraceae	Fruit	Ripe fruits are mixed with salt and eaten fresh	Shuiyan Village, Ma’andi Township	201,511–16	1.9	0.55
*Ficus henryi* Warb. ex Diels	A niao niao xiu	Tree	Moraceae	Fruit	Fruits eaten fresh or liquor brewing	Baohua Township	201,511–40	1.7	0.41
*Ficus hirta* Vahl	Ji zi o si	Shrub	Moraceae	Fruit	Ripe fruits are eaten fresh	Lagu Village, Sanmeng Township	HHD-34	2.0	0.38
*Ficus irisana* Elmer	Qi pu	Tree	Moraceae	Fruit	Ripe fruits are eaten fresh	Xiongjia Village, Adebo Township	HHD-012	1.8	0.39
*Ficus oligodon* Miq.	Xi bo ai xi	Tree	Moraceae	Fruit	Ripe fruits are eaten fresh	Lüshuge Village, Jiayin Township	201,610–02	2.1	0.46
*Ficus pandurata* Hance		Shrub	Moraceae	Fruit, seed	Fruit: eaten fresh; seed: roasted and eaten (kernel)	Lüchun County	201,606–28	2.1	0.48
*Ficus racemosa* L.	A niao niao na	Tree	Moraceae	Fruit, seed	Fruit: eaten fresh; seed: roasted and eaten (kernel)	Lüchun County	201,606–27	2.1	0.51
*Ficus semicordata* Buch.-Ham. Ex Sm.	Hu gan da pa	Tree	Moraceae	Fruit	Ripe fruits are eaten fresh	Lagu Village, Sanmeng Township	HHD-35	2.4	0.44
*Ficus tikoua* Bureau	Wei chao lao e	Liana	Moraceae	Fruit	Ripe fruits are eaten fresh	Xiaoxinjie Township	LB-01	2.0	0.48
*Debregeasia longifolia* (Burm.f.) Wedd.	Mao ma qiang ga	Shrub	Urticaceae	Fruit	Ripe fruits are eaten fresh	Lonajia Village, Jiayin Township	201,506–31	2.1	0.51
*Debregeasia orientalis* C. J. Chen	O ce bu	Shrub	Urticaceae	Fruit, leaf, tender stem	Leaf and stem: potherb; fruit: eaten fresh	Baohua Township	HHD-19	2.5	0.55
*Elatostema involucratum* Franch. and Sav.	Luo bu. a bo	Herb	Urticaceae	Tender stem, leaf	Potherb	Niujiaozhai Township	201,606–13	1.9	0.40
*Gonostegia hirta* (Blume ex Hassk.) Miq.	Pa qian a bo	Herb	Urticaceae	Tender stem, leaf	Potherb	Lüchun County	LB-22	2.1	0.54
*Lecanthus peduncularis* (Wall. ex Royle) Wedd.	A che pa nv	Herb	Urticaceae	Whole plant	Potherb (usually made into soup)	Baohua Township	HHD-30	2.2	0.48
*Castanopsis calathiformis* (Skan) Rehder and E.H.Wilson	A ba a bo	Tree	Fagaceae	Seed	Roasted and eaten (kernel)	Baohua Township	HHD-20	0.8	0.18
*Castanopsis carlesii* var. *spinulosa* W.C.Cheng and C.S.Chao	Che qian a bo	Tree	Fagaceae	Seed	Roasted and eaten (kernel)	Niujiaozhai Township	201,606–10	0.5	0.17
*Castanopsis indica* (Roxb. ex Lindl.) A.DC.	Che si a bo	Tree	Fagaceae	Seed	Roasted and eaten (kernel)	Lüchun County	LB-21	0.8	0.05
*Castanopsis mekongensis* A.Camus		Tree	Fagaceae	Seed	Roasted and eaten (kernel)	Shuiyan Village, Ma’andi Township	201,511–24	0.7	0.20
*Lithocarpus megalophyllus* Rehder and E.H.Wilson	A biu a bo	Tree	Fagaceae	Fruit	Ripe fruits are eaten fresh	Baohua Township	201,511–34	2.8	0.46
*Myricae esculenta* Buch.-Ham. ex D. Don		Tree	Myricaceae	Fruit	Fruits eaten fresh or liquor brewing	Niujiaozhai Township	201,606–06	2.0	0.41
*Gynostemma pubescens* (Gagnep.) C.Y.Wu	Ka kui zha ha	Herb	Cucurbitaceae	Leaf, tender stem	Tea substitute	Lonajia Village, Jiayin Township	201,506–44	0.2	0.18
*Hemsleya macrosperma* C.Y.Wu	A za ku xi	Herb	Cucurbitaceae	Tender leaf	Potherb	Xiongjia Village, Adebo Township	201,506–02	2.5	0.40
*Hodgsonia macrocarpa* (Blume) Cogn.	Zha qi gu lu	Liana	Cucurbitaceae	Seed	Eaten directly, or used for pressing oil	Shuiyan Village, Ma’andi Township	201,511–02	1.9	0.48
*Momordica cochinchinensis* (Lour.) Spreng.	Bei ba na	Liana	Cucurbitaceae	Tender stem, leaf	Potherb	Shuiyan Village, Ma’andi Township	201,511–30	2.9	0.44
*Salacia sessiliflora* Hand.-Mazz.	A ka la ma a bo	Shrub	Celastraceae	Fruit	Ripe fruits are eaten fresh	Lüchun County	201,606–25	2.6	0.41
*Oxalis corniculata* L.	Suan ji cao	Herb	Oxalidaceae	Stem, leaf	Potherb: blanched in boiled water, then soaked in cold water for 2 h	Niujiaozhai Township	201,606–01	2.3	0.47
*Elaeocarpus decipiens* F.B.Forbes and Hemsl.	Na ci ci ha	Tree	Elaeocarpaceae	Fruit	Ripe fruits are eaten fresh	Shuiyan Village, Ma’andi Township	201,511–08	1.9	0.58
*Garcinia cowa* Roxb. ex Choisy	Huang xin shu	Tree	Clusiaceae	Fruit	Ripe fruits are eaten fresh	Baohua Township	201,511–35	2.0	0.47
*Garcinia multiflora* Champ. ex Benth.	Qiu guo a si	Tree	Clusiaceae	Fruit	Ripe fruits are eaten fresh	Niujiaozhai Township	201,606–02	2.4	0.42
*Garcinia xanthochymus* Hook.f. ex T.Anderson	A bu. bu. qie	Tree	Clusiaceae	Fruit	Ripe fruits are eaten fresh	Niujiaozhai Township	201,606–04	2.1	0.52
*Cratoxylum cochinchinense* (Lour.) Blume	Jiu ge ge qia	Tree	Hypericaceae	Tender leaf, young fruit	Tender leaves: tea substitute; young fruit: flavoring agent	Niujiaozhai Township	201,606–11	2.2	0.49
*Cratoxylum formosum* subsp. *pruniflorum* (Kurz) Gogelein	A on a bo	Tree	Hypericaceae	Tender leaf	Tea substitute	Lagu Village, Sanmeng Township	HHD-44	0.9	0.25
*Curculigo capitulata* (Lour.) Kuntze	Ma ni zu se	Herb	Hypoxidaceae	Fruit, tender leaf, tender stem	Fruit: eaten fresh; leaves and stem: potherb	Baohua Township	HHD-18	2.0	0.56
*Curculigo sinensis* S. C. Chen	Mei la pa jia	Herb	Hypoxidaceae	Fruit	Ripe fruits are eaten fresh	Baohua Township	201,511–36	1.9	0.56
*Passiflora wilsonii* Hemsl.	Ba ze	Liana	Passifloraceae	Fruit	Ripe fruits are eaten fresh	Shuiyan Village, Ma’andi Township	201,511–04	2.5	0.51
*Flacourtia ramontchi* L’Hér.	A zi long jie a bo	Shrub	Salicaceae	Fruit	Ripe fruits are eaten fresh, or made into jam, or preserved	Lüshuge Village, Jiayin Township	201,610–10	2.4	0.58
*Baccaurea ramiflora* Lour.	Si suo a si	Tree	Phyllanthaceae	Fruit	Ripe fruits are eaten fresh	Lonajia Village, Jiayin Township	201,506–34	4.2	0.89
*Phyllanthus emblica* L.	Bo can xi ka, xi qia ha	Tree	Phyllanthaceae	Bark, fruit	Fruit: eaten fresh; bark: scraching off the inside tender bark to make dishes	Lüchun County	201,606–29	4.9	0.90
*Rotala indica* (Willd.) Koehne	En ni a bo	Herb	Lythraceae	Tender shoot	Potherb	Lüshuge Village, Jiayin Township	201,610–16	2.2	0.42
*Rotala rotundifolia* (Buch.-Ham. ex Roxb.) Koehne		Herb	Lythraceae	Tender leaf, tender stem	Potherb	Baohua Township	HHD-27	2.1	0.52
*Cleistocalyx operculatus* (Roxb.) Merr. and L.M.Perry		Tree	Myrtaceae	Fruit	Ripe fruits are eaten fresh	Lonajia Village, Jiayin Township	201,506–27	1.6	0.56
*Decaspermum parviflorum* (Lam.) A.J.Scott	A gong gong ni a bo	Tree	Myrtaceae	Fruit	Ripe fruits are eaten fresh	Lonajia Village, Jiayin Township	201,506–28	2.4	0.38
*Syzygium fluviatile* (Hemsl.) Merr. and L.M.Perry	Me ran me xiu na ci a bo	Shrub	Myrtaceae	Fruit	Ripe fruits are eaten fresh	The junction of Lüchun County and Yuanyang County	201,506–09	1.6	0.49
*Syzygium yunnanense* Merr. and L.M.Perry	O ho	Tree	Myrtaceae	Fruit	Ripe fruits are eaten fresh	Lüchun County	201,606–24	2.8	0.45
*Medinilla radiciflora* C.Y.Wu ex C.Chen		Shrub	Melastomataceae	Fruit	Ripe fruits are eaten fresh	Shuiyan Village, Ma’andi Township	201,511–03	2.4	0.39
*Medinilla septentrionalis* (W.W. Sm.) H.L. Li	Qian ben er a si	Shrub	Melastomataceae	Fruit	Ripe fruits are eaten fresh	Niujiaozhai Township	201,606–05	2.8	0.48
*Melastoma affine* D. Don	Bei bai	Shrub	Melastomataceae	Fruit	Ripe fruits are eaten fresh	Xiaoxinjie Township	LB-04	2.2	0.46
*Melastoma normale* D. Don.	Yang er ba cui	Shrub	Melastomataceae	Fruit, leaf	Ripe fruits are eaten fresh	Lüchun County	201,606–20	2.6	0.47
*Osbeckia opipara* C.Y. Wu et C. Chen	Bi ji	Shrub	Melastomataceae	Root, stem	Potherb (usually stewed with meat)	The junction of Lüchun County and Yuanyang County	201,506–11	2.2	0.57
*Canarium album* (Lour.) DC.	Bei le a si	Tree	Burseraceae	Fruit	Ripe fruits are eaten fresh, or preserved	Shuiyan Village, Ma’andi Township	201,511–10	2.3	0.40
*Canarium pimela* K.D.Koenig	Si mo a si	Tree	Burseraceae	Fruit	Ripe fruits are eaten fresh, or preserved	Niujiaozhai Township	201,606–07	2.4	0.44
*Canarium strictum* Roxb.	A bo ma dai	Tree	Burseraceae	Fruit	Ripe fruits are eaten fresh, or preserved	Baohua Township	201,511–42	2.5	0.46
*Choerospondias axillaris* (Roxb.) B. L. Burtt and A. W. Hill	Gei ha a bo	Tree	Anacardiaceae	Fruit	Fruits eaten fresh or liquor brewing	Baohua Township	201,511–41	2.2	0.55
*Dracontomelon duperreanum* Pierre	A zi ren a	Tree	Anacardiaceae	Fruit	Ripe fruits are eaten fresh, or preserved	Lagu Village, Sanmeng Township	HHD-37	2.3	0.49
*Mangifera sylvatica* Roxb.		Tree	Anacardiaceae	Fruit	Ripe fruits are eaten fresh	Qingkou Village, Xinjie Township	201,506–14	2.0	0.40
*Rhus chinensis* Mill.	Ha da da xiu	Tree	Anacardiaceae	Fruit	Preserved fruit	Lonajia Village, Jiayin Township	201,506–42	2.8	0.53
*Arytera littoralis* Blume	Ta mo si song	Tree	Sapindaceae	Shoot	Potherb	Lonajia Village, Jiayin Township	201,506–48	2.6	0.52
*Acronychia pedunculata* (L.) Miq.		Tree	Rutaceae	Fruit	Flavoring agent	Lüshuge Village, Jiayin Township	201,610–11	2.3	0.43
*Tetradium austrosinense* (Hand.-Mazz.) Hartley		Tree	Rutaceae	Fruit	Fruits are edible and used for pressing oil	Xiaoxinjie Township	201,506–07	1.6	0.47
*Zanthoxylum bungeanum* Maxim.	A zao	Tree	Rutaceae	Fruit	Flavoring agent	Xiongjia Village, Adebo Township	HHD-013	2.4	0.48
*Zanthoxylum scandens* Blume		Shrub	Rutaceae	Fruit	Flavoring agent	Lonajia Village, Jiayin Township	201,506–38	2.3	0.43
*Zanthoxylum simulans* Hance		Shrub	Rutaceae	Fruit	Flavoring agent	Lagu Village, Sanmeng Township	HHD-38	2.3	0.44
*Ailanthus altissima* (Mill.) Swingle	Qi la wu ha	Tree	Simaroubaceae	Tender leaf	Potherb	Lonajia Village, Jiayin Township	201,506–50	1.8	0.41
*Bombax ceiba* L.		Tree	Malvaceae	Flower	Potherb	Lagu Village, Sanmeng Township	HHD-49	2.2	0.42
*Microcos nervosa* (Lour.) S.Y.Hu		Tree	Malvaceae	Fruit	Ripe fruits are eaten fresh	Baohua Township	201,511–38	2.3	0.45
*Sterculia brevissima* H.H.Hsue	Sa qiu huo bi	Shrub	Malvaceae	Seed	Roasted and eaten (kernel)	Xiongjia Village, Adebo Township	HHD-017	0.1	0.21
*Sterculia lanceolata* Cav.	Sa qiu huo bi	Tree	Malvaceae	Seed	Roasted and eaten (kernel)	Xiongjia Village, Adebo Township	HHD-016	0.1	0.16
*Sterculia pexa* Pierre	Ni hei gei zi a bo	Tree	Malvaceae	Seed	Stir-fried	Lagu Village, Sanmeng Township	HHD-41	2.5	0.56
*Capparis masaikai* H. Lév.		Liana	Capparaceae	Seed	Natural sweetener	Qingkou Village, Xinjie Township	201,506–20	0.8	0.2
*Crateva unilocularis* Buch.-Ham.	Man nei luo ba	Tree	Capparaceae	Tender stem, leaf	Made into pickles (preserved)	Lüshuge Village, Jiayin Township	201,610–14	3.9	0.75
*Stixis suaveolens* (Roxb.) Pierre		Liana	Capparaceae	Fruit, tender leaf	Fruit: eaten fresh; tender leaves: tea substitute	Lüchun County	201,606–30	2.2	0.42
*Capsella bursa-pastoris* (L.) Medik.	A zu o qi	Herb	Brassicaceae	Tender stem, leaf	Potherb	Lü shuge Village, Jiayin Township	201,610–19	2.7	0.45
*Gynostemma pentaphyllum* (Thunb.) Makino		Herb	Brassicaceae	Tender stem and leaves	Potherb or tea substitute	Lonajia Village, Jiayin Township	201,506–46	2.0	0.49
*Nasturtium officinale* R.Br.	Xi yang cai	Herb	Brassicaceae	Tender leaf	Potherb	Xiaoxinjie Township	LB-10	3.4	0.66
*Rorippa islandica* (Oeder) Borbás		Herb	Brassicaceae	Tender leaf	Potherb (boiled for 5–10 min, then soaked in water to remove pungent taste)	Lagu Village, Sanmeng Township	HHD-53	2.4	0.50
*Erythropalum scandens* Blume	Ha jia ha na bei ying	Liana	Olacaceae	Tender stem, leaf	Potherb	Xiaoxinjie Township	LB-15	2.1	0.52
*Korthalsella japonica* (Thunb.) Engl.	De la	Shrub	Santalaceae	Fruit	Ripe fruits are eaten fresh	Lonajia Village, Jiayin Township	201,506–33	1.7	0.39
*Pyrularia edulis* (Wall.) A. DC.	A ke ke ran a si	Tree	Santalaceae	Fruit	Ripe fruits are stewed or stir-fried	Niujiaozhai Township	201,606–12	2.3	0.50
*Myosoton aquaticum* (L.) Moench	Qian chu a ma	Herb	Caryophyllaceae	Tender leaf, tender stem	Potherb	Xiaoxinjie Township	LB-11	2.2	0.46
*Amaranthus spinosus* L.	Wo zu wo niu	Herb	Amaranthaceae	Tender leaf, tender stem	Potherb	Qingkou Village, Xinjie Township	201,506–21	2.5	0.53
*Amaranthus lividus* L.		Herb	Amaranthaceae	Leaf, stem	Potherb	Lagu Village, Sanmeng Township	HHD-52	2.2	0.47
*Amaranthus viridis* L.	La huo pa ni	Herb	Amaranthaceae	Tender leaf, tender stem	Potherb	Lüshuge Village, Jiayin Township	201,610–23	3.6	0.61
*Chenopodium album* L.	Ge xia wo niu	Herb	Amaranthaceae	Shoot	Potherb	Baohua Township	HHD-28	2.3	0.49
*Phytolacca acinosa* Roxb	Kan bo	Herb	Phytolaccaceae	Leaf	Potherb	Qingkou Village, Xinjie Township	201,506–22	2.3	0.44
*Portulaca oleracea* L.	Yi ca mo ni	Herb	Portulacaceae	Tender leaf, tender stem	Potherb	Lagu Village, Sanmeng Township	HHD-51	1.8	0.52
*Dendrobenthamia hongkongensis* (Hemsl.) Hutch.		Tree	Cornaceae	Fruit	Fruits eaten fresh or liquor brewing	Baohua Township	201,511–39	1.9	0.56
*Dendrobenthamia melanotricha* (Pojark.) W.P.Fang		Tree	Cornaceae	Fruit	Ripe fruits are eaten fresh	Shuiyan Village, Ma’andi Township	201,511–06	2.5	0.47
*Nyssa javanica* (Blume) Wangerin		Tree	Cornaceae	Fruit	Ripe fruits are eaten fresh	Shuiyan Village, Ma’andi Township	201,511–07	2.3	0.44
*Swida macrophylla* (Wall.) Soják		Tree	Cornaceae	Fruit	Used for pressing oil	Xiaoxinjie Township	LB-06	2.0	0.51
*Pouteria grandifolia* (Wall.) Baehni		Tree	Sapotaceae	Fruit	Ripe fruits are eaten fresh	Xiaoxinjie Township	LB-02	1.9	0.52
*Diospyros lotus* L. var. *mollissima* C.Y. Wu		Tree	Ebenaceae	Fruit	Fruits eaten fresh, making liquor or vinegar	Lonajia Village, Jiayin Township	201,506–36	2.4	0.58
*Embelia ribes* Burm.f.		Shrub	Primulaceae	Fruit, shoot	Fruit: eaten fresh; shoot: potherb	Shuiyan Village, Ma’andi Township	201,511–14	2.3	0.42
*Embelia subcoriacea* (C. B. Clarke) Mez		Shrub	Primulaceae	Fruit	Ripe fruits are eaten fresh	Niujiaozhai Township	201,606–03	2.1	0.42
*Maesa montana* A. DC.	Ke tu a bo	Shrub	Primulaceae	Leaf	Tea substitute	Lüchun County	LB-20	0.2	0.39
*Maesa parvifolia* A. DC.		Shrub	Primulaceae	Leaf	Tea substitute	Lüchun County	LB-19	1.4	0.37
*Camellia pitardii* Coh.-St.		Shrub	Theaceae	Petal	Potherb	Lüshuge Village, Jiayin Township	201,610–21	2.7	0.40
*Actinidia kolomikta* (Rupr. and Maxim.) Maxim.	A zi ku nu	Shrub	Actinidiaceae	Fruit	Ripe fruits are eaten fresh	Lonajia Village, Jiayin Township	201,506–32	2.5	0.54
*Saurauia napaulensis* DC.		Tree	Actinidiaceae	Fruit	Ripe fruits are eaten fresh	Baohua Township	201,511–32	2.7	0.58
*Saurauia napaulensis* DC. var. *montana* C. F. Liang and Y. S. Wang		Tree	Actinidiaceae	Fruit	Ripe fruits are eaten fresh	Lüshuge Village, Jiayin Township	201,610–01	2.8	0.39
*Saurauia tristyla* var. *hekouensis* C. F. Liang and Y. S. Wang	A nuo xi	Tree	Actinidiaceae	Fruit	Ripe fruits are eaten fresh	Xiaoxinjie Township	LB-05	2.7	0.42
*Gaultheria leucocarpa* Bl. var. *crenulata* (Kurz) T.Z.Hsu	Xie	Shrub	Ericaceae	Leaf	Potherb (made into soup)	Xiaoxinjie Township	LB-09	2.1	0.48
*Gaultheria longibracteolata* R.C.Fang	Ye lan mei	Shrub	Ericaceae	Fruit	Ripe fruits are eaten fresh	Shuiyan Village, Ma’andi Township	201,511–09	1.9	0.45
*Vaccinium bracteatum* Thunb.	Ha na	Shrub	Ericaceae	Fruit	Ripe fruits are eaten fresh	The junction of Lüchun County and Yuanyang County	201,506–10	2.5	0.55
*Pittosporopsis kerrii* Craib	Ha piao mei che	Shrub	Icacinaceae	Fruit, seed	Fruit: eaten fresh; seed: roasted and eaten (kernel)	Lagu Village, Sanmeng Township	HHD-40	2.3	0.55
*Canthium horridum* Blume	Ha da da nue	Shrub	Rubiaceae	Fruit	Ripe fruits are eaten fresh	Shuiyan Village, Ma’andi Township	201,511–05	2.4	0.51
*Hedyotis tenelliflora* Blume	Gu suo na ci	Herb	Rubiaceae	Whole plant	Potherb (made into soup)	Lagu Village, Sanmeng Township	HHD-42	2.0	0.55
*Amalocalyx yunnanensis* Tsiang		Liana	Apocynaceae	Fruit	Young fruit slices are eaten fresh with the source made by pepper and salt	Lagu Village, Sanmeng Township	HHD-32	2.0	
*Dregea volubilis* (L.f.) Benth. ex Hook.f.	Ku cai	Liana	Apocynaceae	Flower, tender leaf	Potherb	Xiaoxinjie Township	LB-12	3.0	0.72
*Melodinus henryi* Craib	Ke se pa ha	Liana	Apocynaceae	Fruit	Ripe fruits are eaten fresh	Lüshuge Village, Jiayin Township	201,610–06	2.6	0.45
*Lithospermum erythrorhizon* Siebold and Zucc.		Herb	Boraginaceae	Tender stem, leaf	Potherb	Baohua Township	HHD-26	2.0	0.50
*Lycium yunnanense* Kuang and A.M.Lu		Shrub	Solanaceae	tender stem	Potherb	Lonajia Village, Jiayin Township	201,506–47	2.0	0.46
*Solanum nigrum* L.	Wo lun	Herb	Solanaceae	Tender leaf, fruit	Fruit: eaten fresh; tender leaf: potherb	Xiongjia Village, Adebo Township	HHD-014	2.3	0.42
*Solanum torvum* Sw.	Si ma ma ha	Shrub	Solanaceae	Root	Potherb (usually stewed)	Xiongjia Village, Adebo Township	HHD-018	2.9	0.46
*Ligustrum sinense* Lour.	Ci kong ba deng a bo	Tree	Oleaceae	Fruit	Liquor brewing	Lagu Village, Sanmeng Township	HHD-46	0.6	0.05
*Rhynchotechum obovatum* (Griff.) B.L. Burtt		Shrub	Gesneriaceae	Fruit	Ripe fruits are eaten fresh	Lüshuge Village, Jiayin Township	201,610–03	1.7	0.39
*Plantago asiatica* L.	Ka pae ca	Herb	Plantaginaceae	Whole plant	Potherb	Xiongjia Village, Adebo Township	201,506–01	2.3	0.51
*Plantago asiatica L. subsp*. *erosa* (Wall.) Z. Y. Li	Ka pae ca	Herb	Plantaginaceae	Tender leaf	Potherb (usually stewed or made into soup)	Baohua Township	HHD-22	2.8	0.53
*Plantago depressa* Willd.	Ha pa yu cai	Herb	Plantaginaceae	Whole plant	Potherb (soaked in water and sir-fried)	Xiaoxinjie Township	LB-08	2.3	0.55
*Mayodendron igneum* (Kurz) Kurz	A ci ma ha nen	Tree	Bignoniaceae	Flower	Potherb	Lüchun County	LB-23	2.8	0.43
*Clinopodium chinense* (Benth.) Kuntze	Zhaun zhuan cai	Herb	Lamiaceae	Tender leaf, tender stem	Potherb	Lagu Village, Sanmeng Township	HHD-50	2.4	0.44
*Mentha canadensis* L.	Wo zhi zhi ma	Herb	Lamiaceae	Tender leaf, tender stem	Flavoring agent	Shuiyan Village, Ma’andi Township	201,511–11	4.6	0.75
*Rabdosia coetsoides* C.Y.Wu	Nu ha ma	Herb	Lamiaceae	Whole plant	Tea substitute or cooked with meat (Potherb)	Shuiyan Village, Ma’andi Township	201,511–15	0.7	0.23
*Helwingia japonica* (Thunb.) F.Dietr.	Huo tie tie du	Shrub	Helwingiaceae	Tender stem, leaf	Potherb (blanched in hot water, then soaked in cold water before cooking)	Niujiaozhai Township	201,606–18	1.9	0.53
*Campanumoea javanica* Blume	A mi nan guo	Liana	Campanulaceae	Fruit	Ripe fruits are eaten fresh	Lagu Village, Sanmeng Township	HHD-36	1.6	0.56
*Lobelia angulata* G.Forst.		Herb	Campanulaceae	Tender leaf, tender stem	Potherb	Shuiyan Village, Ma’andi Township	201,511–31	1.8	0.42
*Adenocaulon himalaicum* Edgew.	Bu lü wu hu	Herb	Asteraceae	Tender leaf	Potherb	Niujiaozhai Township	201,606–15	1.8	0.45
*Bidens pilosa* L.	Hei ni zuo ge mo	Herb	Asteraceae	Tender leaf	Potherb (stewed until it is tender)	Qingkou Village, Xinjie Township	201,506–26	2.0	0.53
*Cirsium japonicum* (Thunb.) Fisch. ex DC.	Che pei a gong	Herb	Asteraceae	Root	Stewed with pork for nourishing	Baohua Township	HHD-21	2.9	0.43
*Crassocephalum crepidioides* (Benth.) S. Moore	O mi o sa	Herb	Asteraceae	Tender leaf	Potherb	Baohua Township	HHD-29	3.9	0.76
*Eclipta prostrata* (L.) L.	A ji mei, a ge wo chi	Herb	Asteraceae	Tender leaf	Potherb	Qingkou Village, Xinjie Township	201,506–25	2.2	0.49
*Gnaphalium affine* D. Don	A mi sha chu	Herb	Asteraceae	Leaf, stem	Potherb	Qingkou Village, Xinjie Township	201,506–23	2.1	0.42
*Ixeris polycephala* Cass.		Herb	Asteraceae	Tender leaf, tender stem	Potherb (blanched before eating and making salad)	Lüchun County	LB-26	3.9	0.75
*Lactuca serriola* L.		Herb	Asteraceae	Tender leaf, tender stem	Potherb	Xiaoxinjie Township	LB-13	2.8	0.52
*Lagedium sibiricum* (L.) Soják	E si lao gong zi	Herb	Asteraceae	Tender leaf, tender stem	Potherb	Shuiyan Village, Ma’andi Township	201,511–29	2.8	0.54
*Laggera pterodonta* (DC.) Sch.Bip. ex Oliv.	Wo sa la ma	Herb	Asteraceae	Whole plant	Potherb (blanched in hot water, then soaked in cold water before cooking)	Lüchun County	LB-25	2.9	0.51
*Viburnum dilatatum* Thunb.	Pu tong a bo	Shrub	Adoxaceae	Fruit	Fruits eaten fresh or liquor brewing	Qingkou Village, Xinjie Township	201,506–16	2.7	0.51
*Dipsacus asperoides* C.Y.Cheng and T.M.Ai	Pao tou cao	Herb	Caprifoliaceae	Tender leaf, root	Potherb (Usually stewed with pork or made into soup)	Baohua Township	HHD-23	2.7	0.51
*Valeriana jatamansi* Jones	Ye zuo zuo pu	Herb	Caprifoliaceae	Flower, root	Flower: eaten fresh (Potherb); root: stewed for nourishing	Lonajia Village, Jiayin Township	201,506–40	1.7	0.51
*Acanthopanax trifoliatus* (L.) Voss	Jiu duo	Shrub	Araliaceae	Tender stem	Potherb	Lüchun County	LB-24	2.5	0.50
*Eleutherococcus senticosus* (Rupr. et Maxim.) Maxim.		Shrub	Araliaceae	Tender stem, leaf	Potherb	Lüshuge Village, Jiayin Township	201,610–18	2.3	0.42
*Centella asiatica* (L.) Urb.	Ban chao wo ba	Herb	Apiaceae	Tender leaf, tender stem	Potherb	Shuiyan Village, Ma’andi Township	201,511–27	3.0	0.58
*Eryngium foetidum* L.	Ga la ya so	Herb	Apiaceae	Tender leaf, tender stem	Flavoring agent or stir-fried (potherb)	Qingkou Village, Xinjie Township	201,506–18	1.9	0.52
*Ligusticum chuanxiong* S.H.Qiu, Y.Q.Zeng, K.Y.Pan, Y.C.Tang, and J.M.Xu	Tong e jian sa	Herb	Apiaceae	Tender leaf	Flavoring agent or stir-fried (potherb)	Qingkou Village, Xinjie Township	201,506–17	2.6	0.53
*Oenanthe javanica* (Blume) DC.	Zha suo	Herb	Apiaceae	Tender leaf, tender stem	Potherb	Niujiaozhai Township	201,606–14	4.3	0.82
*Sanicula astrantiifolia* H. Wolff ex Kretschmer	Xiao hei yao	Herb	Apiaceae	Whole plant	Potherb (usually sir-fried)	Lonajia Village, Jiayin Township	201,506–54	2.9	0.48
Pteridophyta									
*Lygodium digitatum* C. Presl	Ha da da xiu	Liana	Lygodiaceae	Tender stem	Potherb	Lüshuge Village, Jiayin Township	201,610–17	2.5	0.54
*Pteridium aquilinum* var. *latiusculum* (Desv.) Underw. ex A. Heller	Ye qie	Herb	Dennstaedtiaceae	Shoot	Potherb	Shuiyan Village, Ma’andi Township	201,511–26	2.7	0.45
*Ceratopteris thalictroides* (L.) Brongn.	Da lie	Herb	Pteridiaceae	Shoot	Potherb	Niujiaozhai Township	201,606–17	2.6	0.47
*Callipteris esculenta* (Retz.) J. Sm. ex T. Moore and Houlston		Herb	Athyriaceae	Tender leaf	Potherb	Lagu Village, Sanmeng Township	HHD-47	2.5	0.56
*Callipteris esculenta* var. *pubescens* (Link) Ching		Herb	Athyriaceae	Tender leaf	Potherb	Baohua Township	HHD-24	1.8	0.56
*Gymnocarpium remotepinnatum* (Hayata) Ching	Ha	Herb	Athyriaceae	Tender leaf	Potherb	The junction of Lüchun County and Yuanyang County	201,506–12	2.1	0.58
*Parathelypteris glanduligera* (Kunze) Ching	Ha da	Herb	Thelypteridaceae	Shoot	Potherb	Lonajia Village, Jiayin Township	201,506–49	2.7	0.56
*Marsilea quadrifolia* L.	He dou a ya mo	Herb	Marsileaceae	Tender leaf	Potherb	Shuiyan Village, Ma’andi Township	201,511–25	3.3	0.59

The order of plant species in this table is followed by the APG IV system, gymnosperms classification system (1978), and Qinrenchang fern plant classification system (1978)

**Table 4:** Life forms of WEP species in Honghe terraced rice paddy system

Life form	Records	Percentage
Herbs	79	35.3%
Trees	68	30.4%
Shrubs	46	20.5%
Lianas	21	9.4%
Bamboo	10	4.5%

All WEP species were also classified by their edible parts (Table 5). The recorded edible parts of WEP species included the whole plant, root, stem and leaf, flower, fruit, seed and shoot, and other parts like bark and tuber. For several WEP species, like *Bauhinia acuminata* var. *candida* and *Senna tora*, multiple parts can be consumed. These results embody the diversity of edible parts of WEP species in Honghe terraced rice paddy fields and indicate that local people have become well adapted to the local environment for centuries. The various uses and preparation methods are recorded in Table 6.

**Table 5:** Edible parts of WEP species in Honghe terraced rice paddy system

Part used	Records	Percentage
Fruit	98	43.8%
Stem and leaf	83	37.1%
Shoot	18	8.0%
Seed	18	8.0%
Flower (petal, bract)	16	7.1%
Whole plant	8	3.6%
Root	6	2.7%
Rhizome	3	1.3%
Tuber	3	1.3%
Bark	1	0.4%

**Table 6:** Preparation and uses of WEP species in Honghe terraced rice paddy system

Preparation and uses	Records	Percentage
Potherb	95	42.4%
Eaten fresh	84	37.5%
Flavoring agent	16	7.1%
Nuts	12	5.4%
Tea substitute	11	4.9%
Bamboo shoots	10	4.5%
Liquor brewing	8	3.6%
Grain substitute	2	–
Special tofu	2	–
Sweetener	1	–

The plant stems and leaves are also collected widely (Table 6), and these are mostly consumed as a potherb, which is generally referred to as “wild vegetables” locally. The Hani people usually consume potherbs by stir-frying or by boiling them in a soup. The blended vegetables in soups are usually mixed with natural spices before eating. There are 16 species with edible flowers (Table 5), including *Musa itinerans* (bracts only). These edible flowers could be an essential source of nutrition for local people. It has been previously reported that edible flowers are rich in nutrients and micronutrients and that some of their extracts are useful as medicines [30–32]. Potherb is the most consumed group (Table 6) of WEP species in Honghe with 75 species (33.5%). In China today, wild vegetables, or “ye cai,” have become popular food products that are increasingly being served in restaurants due to their flavor and a widespread perception of their superior nutritional values [33]. In the Honghe area, wild vegetables also play a vital role in local livelihood as food and dietary supplements. These wild vegetables are mainly collected in the mountains above the rice paddy fields and forest lands, and the collection time lasts from January to October but mainly occurs in the spring. Some plants, like *Houttuynia cordata* and *Oenanthe javanica*, can be collected throughout the year.

According to Table 5, edible fruit is the most popular group (98 species, 43.8%). These are usually consumed freshly without processing, which is the second most common food preparation method for the Hani WEP species (Table 6). Also, fruits can be consumed in several different ways. For example, *Amomum maximum* fruits are used locally as a natural spice that can help infirm people regain their appetite; the fruits of *Ligustrum sinense* are used by the local Hani people to brew a unique alcoholic drink, and *Canarium album*’s fruits can be preserved into pickles.

Ten species of bamboo shoots can be made into different dishes that are high in nutritious fibers. Some WEP species in the Honghe region can also be used as natural flavoring agents (16 species), nuts (12), tea substitutes (11), liquor-brewing ingredients (8), grain substitutes (2), and special tofu (2). Exceptionally, there is only one species, *Capparis masaikaii,* that is used as a natural sweetener by local communities. The locals usually remove the seed coat and chew the kernel directly. *C. masaikaii* contains high levels of mabinlin, a sweet protein with 400 times the sweetness of sucrose but with meager calories, and consequently, this plant has a high potential for future application in the food industry [34].

### Two special cases of WEP species

**Fig. 4:**
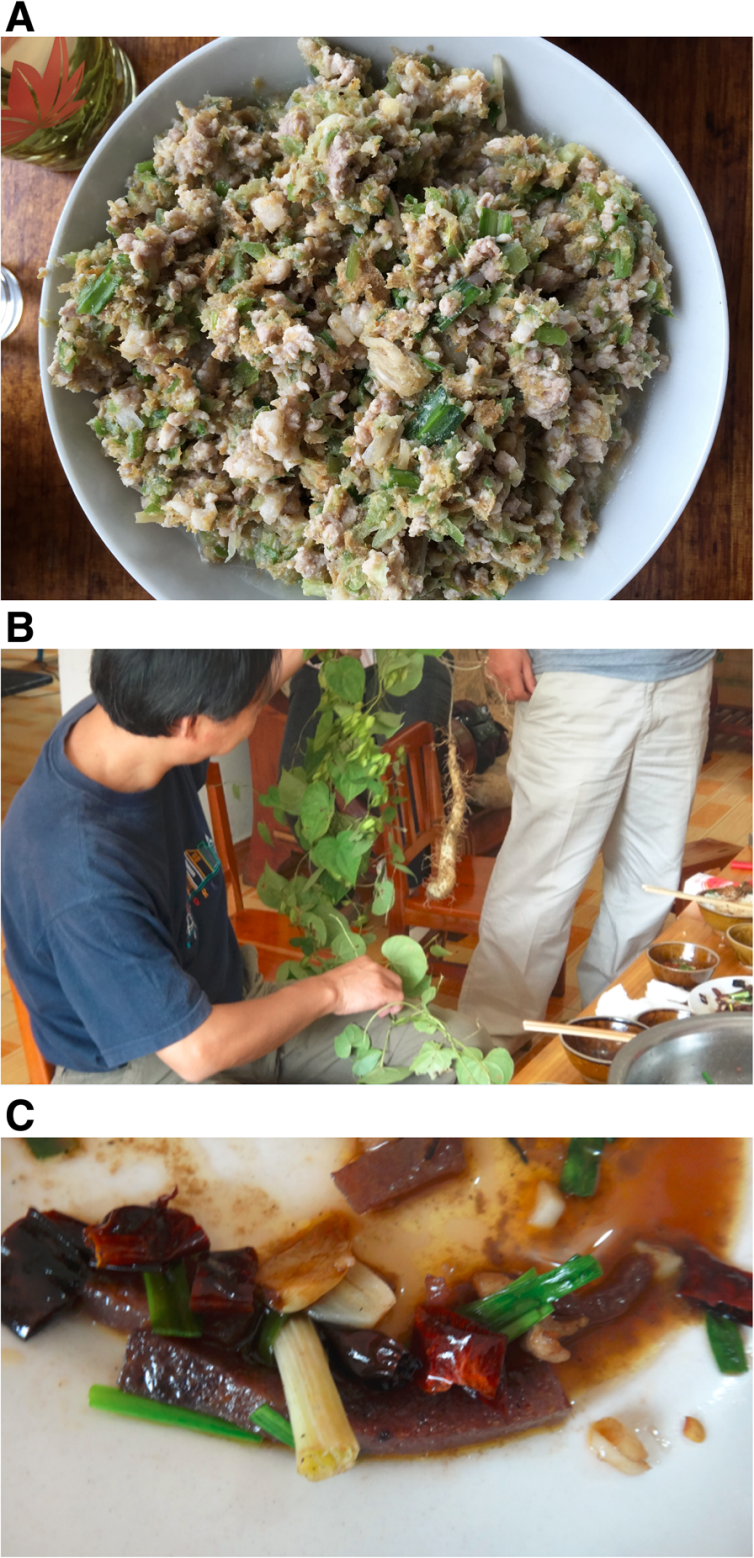
Dish of *Phyllanthus emblica* (**a**). *Dioscorea subcalva* and dish of *D. subcalva* (**b**, **c**)

During our investigation, some unique cases of utilizing and processing WEP species were observed. In the Honghe area*, Phyllanthus emblica* bark is prepared in an unusual way (Fig. 4a). Local Hani people collect the *P. emblica* from mountainous forests, remove the branches, and peel off the outer layer of bark, grating off the bitter-tasting inner bark by using pottery shards. Traditionally, they adjust the bitter taste by mixing rice porridge paste with the tender inner bark. Then, the grated inner bark is mixed with roasted ribs, sliced pork liver, salt, and spices and eaten as a traditional dish. Besides its culinary use, *P. emblica* is also used medicinally for its potential anti-microbial, antioxidant and anti-tumor, hypolipidemic, hypoglycemic, and antihypertensive properties [35].

The traditional preparation of *Dioscorea subcalva* in cuisine is also distinctive. Local women first peel the thin skin from the *D. subcalva* tubers (Fig. 4). They then use a special tool to grate the peeled tubers into a container with a hot water-ash solution. When the solution has cooled, all of the grated tubers congeal into a sticky and elastic clump. These clumps can be cut into slices and stir-fried with meat. In another use of *D. subcalva,* local people scrape off the exudate from its tuber and apply this exudate directly onto wounds for wound healing [36, 37]. The exudate has high polysaccharide content and possesses good antioxidant bioactivity [36, 37].

### The UV and FUI value of WEP species in the Honghe area

Quantitative analyses were calculated to determine the local importance of each wild edible species. The use values (UV) and frequency of utilization indices (FUI) of each species were calculated. The 20 WEP species with the highest UV are listed along with their average FUI in Table 7.

**Table 7:** Top 20 WEP species with highest use value in Honghe terraced rice paddy system

Scientific name	Preparation and uses	FUI	UV
*Litsea pungens*	Flavoring agent	4.9	0.92
*Phyllanthus emblica*	Fruit: eaten fresh; bark: special dishes	4.9	0.90
*Baccaurea ramiflora*	Ripe fruits are eaten fresh	4.2	0.89
*Houttuynia cordata*	Potherb or flavoring agent	4.5	0.89
*Oenanthe javanica*	Potherb	4.3	0.82
*Colocasia gigantea*	Potherb (cooked thoroughly)	4.0	0.80
*Crassocephalum crepidioides*	Potherb	3.9	0.76
*Crateva unilocularis*	Made into pickles (preserved)	3.9	0.75
*Mentha canadensis*	Flavoring agent	4.6	0.75
*Ixeris polycephala*	Potherb	4.0	0.75
*Zingiber striolatum*	Potherb	3.0	0.73
*Dregea volubilis*	Potherb	3.0	0.72
*Musa acuminata*	Fruit: eaten fresh; flower and pith part: potherb	3.8	0.70
*Ficus auriculata*	Ripe fruits are eaten fresh	3.4	0.68
*Nasturtium officinale*	Potherb	3.4	0.66
*Acacia pennata*	Potherb	3.4	0.65
*Elatostema involucratum*	Potherb	3.3	0.65
*Amaranthus viridis*	Potherb	3.6	0.61
*Marsilea quadrifolia*	Potherb	3.3	0.59
*Centella asiatica*	Potherb	3.0	0.58

*Litsea pungens* had the highest UV and average FUI (Table 7). Because of its unique flavor and positive effects on human health, it has become the most commonly used edible species as a spice. Some local people even use the oil extracted from this species to repel mosquitos. Based on both local medical theory and scientific research, *L. pungens* can help to promote appetite and improve digestion [38]. *Mentha canadensis* is also a popular spice among local people, especially for cooking meat. However, its average FUI value and UV are relatively lower than for *L. pungens*. The second highest UV belongs to *Phyllanthus emblica,* while its average FUI value is similar to that of *L. pungens*. The high UV and average FUI values of *P. emblica* may be attributed to its juicy and tasty fruits and its special cultural property: its tender bark is consumed in every October Festival and God Walled Festival.

Several other plants were found to be popular as wild vegetables in our study area based on their high UV and average FUI, including the following: *Acacia pennata*, *Ixeris polycephala*, *Amaranthus viridis*, *Centella asiatica*, *Colocasia gigantea*, *Crassocephalum crepidioides*, *Dregea volubilis*, *Elatostema involucratum*, *Houttuynia cordata*, *Oenanthe javanica*, and *Zingiber striolatum*. For example, *H. cordata* is the most common wild vegetable in Southern China. Many modern scientific studies have indicated that this species possesses excellent anti-microbial, anti-cancer, anti-inflammatory, and immuno-enhancement bioactivities [39, 40]. *C. gigantea* is also a popular food plant, especially in Southwest China, and we found that the stem of *C. gigantean* contains high levels of nutrients and no heavy metals [41]. In addition, the inflorescence of *Musa acuminata* and *Zingiber striolatum* and the fruits of *Ficus auriculata*, *Baccaurea ramiflora*, and *Musa acuminata* are all relatively popular and important in local daily life.

### The effect of WEP species on communities’ economic income

Based on our interviews with key informants in local markets, the trading volume of WEP species was on a very small scale (less than 15 yuan each stall), which means selling WEP species could only bring a small income supplement and was usually unstable for local people. Therefore, driven by economic profits, many villagers have switched from growing rice to other economic plants like bananas in Hani terraced rice paddy fields. Many Hani informants reported that planting bananas can bring more income than growing rice. While in the short term, Hani farmers can get a higher income by growing bananas; they have to dry the rice paddy fields before planting bananas, which is against the Hani traditional ideas of sustainability. In doing so, the original construction and wetland habitats, as well as biodiversity, will be destroyed in the long term. Nevertheless, according to our observations, drying rice paddy to plant others was shown in different places in the Honghe region, especially in Jinping County.

### The sources of and threats to the diversity of WEP species in the Honghe region

**Fig. 5:**
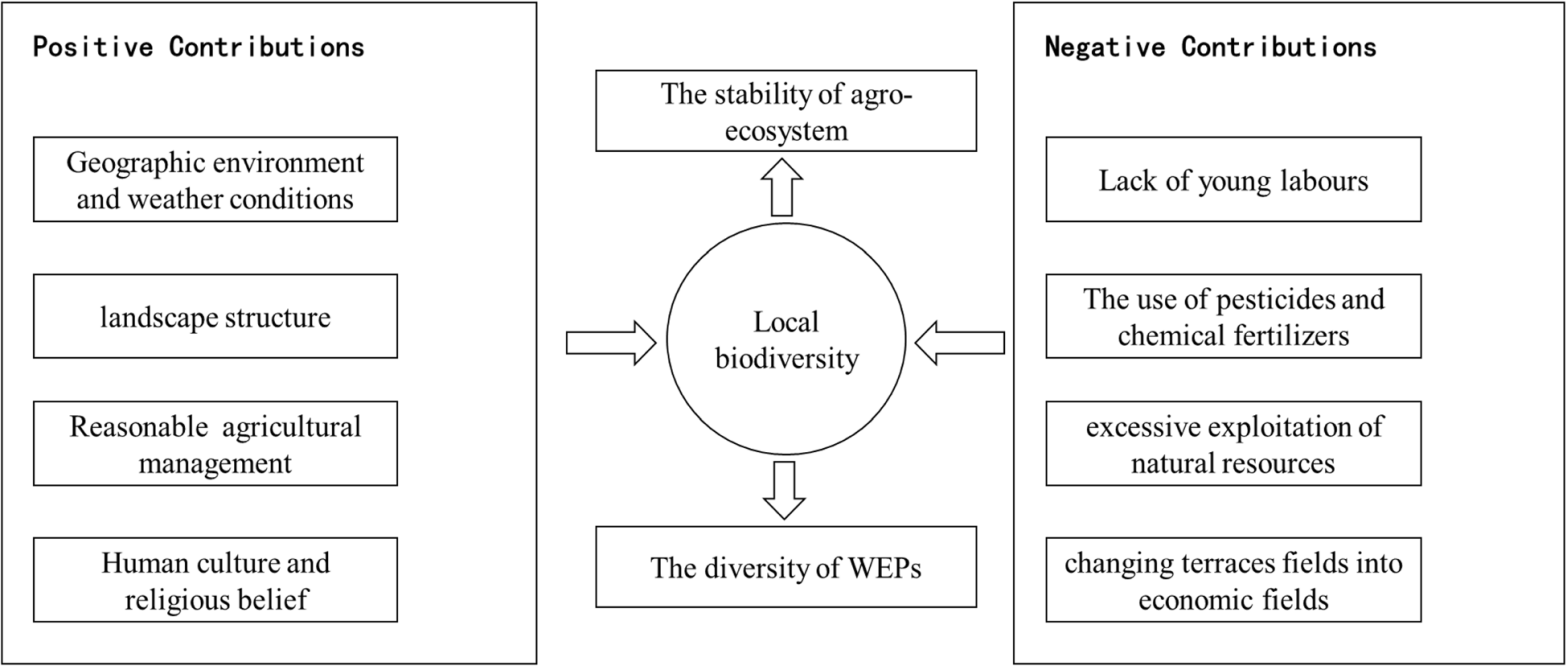
The relationship between local biodiversity and its effect factors

Our investigation revealed the diversity of WEP species in the Honghe area. Several reasons contributing to local biodiversity and WEP diversity have been analyzed (Fig. 5). The varied natural geographic environments and weather conditions are two of the main reasons for the diversity of WEP species. Secondly, the sustainable landscape structure constructed by the locals, including the four critical elements of forests, villages, terraces, and rivers, has a robust regulating ability, particularly for the regulation of essential water resources. Additionally, with the guidance of abundant traditional knowledge concerning reasonable agricultural management, excellent ecological benefits have been made to improve and maintain stability and biodiversity in the whole agroecosystem. Some Hani taboos, village regulations, and non-governmental agreements, as well as local religious beliefs like the worship of the mountain deity and magic woods, have restrained the behaviors of local people and protected the surroundings.

However, the stability of Hani terraced rice paddy fields, which has been maintained for centuries, is now facing a series of challenges (Fig. 5). In our research area, traditional rice planting methods have been damaged by modernization. Based on our investigation, local young people prefer to work in urban areas to make more money instead of doing farm works in their hometowns. Traditional knowledge related to farmland management is only mastered by the older generation and is fading away rapidly (Table 3), and 18% of recorded species lack local Hani names. One reason for this lack of local names is that the Hani people are traditionally illiterate so that traditional knowledge can only be passed on orally by generations, so this knowledge is vulnerable to loss via acculturation. Although a writing system of the Hani language has been in place since 1957, it has not been widely adopted in the Hani communities. In addition, large-scale growing of hybrid rice requires less use of traditional agricultural methods and instead relies on pesticides and chemical fertilizers. Furthermore, the excessive exploitation of natural resources and the drying and changing of traditional rice terraces fields into economic fields are becoming increasingly frequent in the Honghe area nowadays. These phenomena are leading to a sharp decrease in the diversity of traditional knowledge and local biodiversity, which might damage the agroecosystem in this region [42, 43].

Since 2013, when the Hani agroecosystem was elected into the UNESCO World Heritage List, tourism has increased markedly. In 2015, the tourism industry generated about 191.5 billion yuan and accounted for about 70% of local government revenues (out of 275.6 billion yuan). However, the local villagers have obtained minimal economic benefits from the local tourism industry. The traditional agroecosystem cannot sustain the daily food needs of the local people anymore. The Hani are now turning to the tourism industry, which may help to protect the traditional knowledge and biodiversity in this agroecosystem.

## Conclusion

An ethnobotanical study on WEP species from the Hani terraced rice paddy agroecosystems in Southeast Yunnan, China, was conducted. Two hundred and twenty-five species (belonging to 170 genera and 90 families) of wild edible species and the information of their life forms, edible parts, and preparation methods were documented. Based on our analysis, the most widely eaten parts of WEP species are fruits, stem, and leaves. The most common processing methods for WEP species are cooking them as a potherb or eating them fresh. These results are closely related to the local lifestyle and reflect the local biodiversity. The use values (UV) of WEP species were also calculated, and the 20 species with the highest use value were listed. Compared with other WEP species, these 20 species are relatively more important to local daily life, and *Litsea pungens*, a local common natural spice, is the most popular WEP based on its high UV metric.

The reasons for local biodiversity and the challenges for local agroecosystem have been analyzed. This agroecosystem is facing severe problems concerning natural resource conservation, environmental protection, and the economic development of local communities in this agroecosystem. Prestigious designations like UNESCO World Heritage Site have helped to promote ecotourism, which has begun to improve the livelihood of local people while sustaining the operation of this agroecosystem.

In conclusion, there are abundant plant resources in the Hani terraced rice paddy field system because it is an ancient sustainable agroecosystem. However, in modern times, this region has suffered a series of threats. It is, therefore, critical to develop an effective way to protect it and to ensure its sustainability for its inhabitants.

## Data Availability

All data generated or analyzed during this study are included in this published article (and its supplementary information files).
